# Trial for Malpractice

**Published:** 1845-07

**Authors:** 


### TRIAL FOR MALPRACTICE.

*Wm. Tims* vs. *James P. White.*—This was an action brought in the Circuit Court, in Erie co., N. Y., and tried on the 18th June, ult. The material facts of the case, as reported, are: that the plaintiff fractured the femur (the date of the accident, and the point of fracture do not appear); Dr. White dressed it, using the double inclined plane; that at the end of forty-seven days the apparatus was removed, the patient being directed to remain in bed; there was some pain; patient walked about, and, at the end of a certain time, the limb was found to be an inch shorter than the other, and angular at the point of fracture. Dr. Wilcox testified, that at the time the dressing was removed, the fracture appeared to be consolidated, and "the limbs were of equal length and proper direction." Drs. Bissel and Flint testified, that the patient said his leg was straight when the splints were taken off. Most of the medical testimony was to the effect that the treatment was judicious; that the double inclined plane is the preferable apparatus; that it may be removed in some cases as soon as forty days after its application. It was proved that, in this case, there was much pain and spasmodic action after the removal of the dressings. The jury did not agree upon a verdict, this being the second time the same result had been obtained.

*Remarks.*—If we place implicit reliance on the testimony in this case, we must believe that the deformity and shortening of the member took place after the removal of the splints; that this resulted either from the improper use of the limb, or from spasmodic action of the muscles. In either case there can be little doubt that the verdict should have been in favor of the defendant.

We were, however, a little surprised, that the eminent and judicious men called as medical witnesses, should have given such favorable opinions of the double inclined plane, in fracture of the *shaft of the femur*. That it is preferable in fractures occurring near the extremities of that bone, we do not doubt; but, from observation, and personal experience with both kinds, we must give a preference, in fractures near the extremities, to the straight apparatus. The result of this case, and the testimony given in it, do but confirm us in this opinion. Thus, Prof. Hamilton, who uses the angular apparatus, says, with a frankness worthy of all praise, he "never succeeded in making a fractured limb of the same length as a well one." For ourselves, since using the straight splint of Dessault, three cases of fracture of the thigh, near its middle, have come under our care, and in neither of these has there been, so far as the patient could discern, the slightest difference between the members of the injured and well sides. We say as far as the patient could discern, for accurate measurement with a string was not made. But in no case, in the adult, have we removed it before the twelfth week.

In a great number of cases, however, owing to the character of the injury, the irritability of the muscles, the state of the system which retards or prevents the formation of callus, the indocility or want of care on the part of the patient, it is impossible for the most skilful surgeon, with the most perfect apparatus, to reproduce a perfect limb; and it is important that this should be generally known, as it would, in many instances, protect surgeons from prosecutions originating in improper motives, personal feeling, or ignorance, whether they be found in patients, professional men, or the public.

