# Correction

**Published:** 1845-08

**Authors:** 


CORRECTION.

We have received from Prof. Hamilton, a letter in relation to our remarks on the trial for malpractice, (Timms *v.* White,) in the July number of this journal. In the report of that trial published in the "Buffalo Pilot," Dr. Hamilton is made to say, that he never succeeded in making a fractured limb of the same length as a well one; whereas, the words he used were—"I have never succeeded in an *oblique* of the *thigh*, occurring in an *adult*, to make the limb of the same length as before the fracture."

We would also correct a misprint in our notice of that trial. Instead of saying that the straight apparatus is preferable in fractures occurring near the extremities of the femur, it should be near the middle of that bone. We learn also, by the letter, that such is the opinion of Prof. Hamilton. D. B.

